# Curriculum-reinforcement learning on simulation platform of tendon-driven high-degree of freedom underactuated manipulator

**DOI:** 10.3389/frobt.2023.1066518

**Published:** 2023-07-12

**Authors:** Keung Or, Kehua Wu, Kazashi Nakano, Masahiro Ikeda, Mitsuhito Ando, Yasuo Kuniyoshi, Ryuma Niiyama

**Affiliations:** ^1^ School of Science and Technology, Meiji University, Kawasaki, Japan; ^2^ Graduate School of Information Science and Technology, The University of Tokyo, Tokyo, Japan; ^3^ College of Information Science and Engineering, Ritsumeikan University, Shiga, Japan

**Keywords:** reinforcement learning, curriculum learning, simulation, tendon-driven system, underactuated manipulator, soft robotics, bio-inspired robot

## Abstract

A high degree of freedom (DOF) benefits manipulators by presenting various postures when reaching a target. Using a tendon-driven system with an underactuated structure can provide flexibility and weight reduction to such manipulators. The design and control of such a composite system are challenging owing to its complicated architecture and modeling difficulties. In our previous study, we developed a tendon-driven, high-DOF underactuated manipulator inspired from an ostrich neck referred to as the Robostrich arm. This study particularly focused on the control problems and simulation development of such a tendon-driven high-DOF underactuated manipulator. We proposed a curriculum-based reinforcement-learning approach. Inspired by human learning, progressing from simple to complex tasks, the Robostrich arm can obtain manipulation abilities by step-by-step reinforcement learning ranging from simple position control tasks to practical application tasks. In addition, an approach was developed to simulate tendon-driven manipulation with a complicated structure. The results show that the Robostrich arm can continuously reach various targets and simultaneously maintain its tip at the desired orientation while mounted on a mobile platform in the presence of perturbation. These results show that our system can achieve flexible manipulation ability even if vibrations are presented by locomotion.

## 1 Introduction

Manipulators with redundant degrees of freedom (DOFs) are beneficial for representing various postures as the end-effectors reach a specific position and orientation (Chirikjian and Burdick, 1994; Tatlicioglu et al., 2009; Baur et al., 2012). This feature is characterized by the contribution of additional joints toward higher dexterity in the interior of their workspace, which is often considered a solution for obstacle scenarios (Gong et al., 2016; Xu et al., 2019). However, an increase in the number of actuators for such redundant joints leads to anincrease in the weight along the arm. This increases energy consumption, safety, and tip-over problems when the manipulator is mounted on a mobile platform. The use of under-actuated joints with a tendon-driven system is considered a solution to these problems (Chung et al., 1998), with benefits such as (1) providing flexibility in manipulation, (2) reducing the weight of the arm, and (3) placing the center of mass of the manipulator close to the mobile platform. In our previous work, we proposed a tendon-driven high-DOF underactuated manipulator inspired by the vertebral column of an ostrich, referred to as the Robostrich arm (Mochiyama et al., 2022). However, the inverse kinematics of such a complicated system are too complex to be fully known. This leads to difficulty in controlling the Robostrich arm using traditional model-based control methods, even for simple reach tasks.

Recently, researchers have applied reinforcement learning to manipulator tasks. Reinforcement learning is a trial-and-error-based approach that allows an agent to automatically acquire skills as it interacts with its environment, and is therefore often effective for manipulation tasks. Among these tasks, reaching has been the main subject of studies on the combination of redundant arms and reinforcement learning. Morimoto et al. added a payload to the arm to reach a stick (Morimoto et al., 2021), Satheeshankar et al. studied redundant manipulators that could reach multiple target points (Satheeshbabu et al., 2020). However, the motion of a tendon-driven manipulator is often a complex and nonlinear time-varying system, which is difficult to achieve simply using reinforcement learning methods. In such complex scenarios, it is challenging to design advanced reinforcement-learning techniques for complicated manipulation tasks. In this study, we aimed to address the aforementioned problems regarding design and control issues.

Inspired by the fact that the learning process for humans and animals generally follows an order from easy to difficult, we focus on the concept of curriculum learning (Bengio et al., 2009). Compared with the general paradigm of indiscriminate machine learning, curriculum learning imitates the process of human learning, advocating that the model should start learning from easy samples and gradually progress to complex samples and knowledge, further showing the two advantages for learning. The first is that it can accelerate the training of machine learning models; under the condition that it achieves the same model performance, curriculum learning can speed up training and reduce the number of training iteration steps. The second advantage is that the model can obtain better generalization performance, that is, the model can be trained to a better local optimum state.

On the other hand, constructing simulations often benefits researchers who design robots, helping to confirm the robot’s motion and train it. Software such as Gazebo, CoppeliaSim (previously V-Rep), Multi-Joint dynamics with Contact (MuJoCo), and Matlab have been used to simulate a robot in many studies (Xiao et al., 2017; Huang et al., 2020; Shahid et al., 2021; Rooban et al., 2022). However, in contrast to conventional manipulators with direct motor-driven joints, the relationship between tendons and joints change must be closely considered.

This study particularly focuses on control and simulation problems of a tendon-driven high-DOF underactuated manipulator. A curriculum-reinforcement learning framework was proposed for controlling a complicated manipulator to accomplish complex manipulation tasks. In addition, an approach was addressed to simulate the tendon-driven system for constricting the training environment. We first confirmed the motion of the tendon-driven manipulator using the developed simulator. Next, the workspace of the Robostrich arm was investigated. Simultaneously controlling position and orientation is a challenging task for such a high-DOF underactuated manipulator, in particular when the manipulator is composed of a tendon-driven system. We compared the proposed learning method with conventional Soft Actor-Critic (SAC) learning in a reaching task using the Robostrich arm. Consequently, two application tasks were conducted to investigate the robustness of our method in the presence of noise, which is caused by the locomotion of the mobile base, providing interaction with the environment: (1) stabilize the tip movement during walking; (2) track a set of sub-target positions to pass through a narrow gap and reach the final target position at the top.

The contributions of this study are as follows.

• A Curriculum-reinforcement learning approach is proposed with the learning lesson definitions depending on the complexities. These lessons enable a manipulator with complicated structure to gradually process from simple to more complex tasks.
• A simulator has been developed using MuJoCo to study tendon-driven underactuated manipulators, which can be used for tendon-driven systems whose structures are too complicated to be accurately modeled.
• We demonstrate through simulation that the complicated manipulator can learn to accomplish complex tasks, even under perturbations when the manipulator was mounted on a mobile platform.

The remainder of this paper is organized as follows. In Section 2, related works are addressed. Section 3 introduces the proposed framework of curriculum-reinforcement learning and corresponding lesson definitions. Section 4 describes the simulation environment, experimental setups, and investigation of properties of the Robostrich arm movement. Section 5 presents the simulation results of the proposed learning method. Section 6 presents the conclusions and future work.

## 2 Related works

### 2.1 Curriculum with reinforcement learning

Curriculum learning is widely used in machine learning frameworks, especially in tasks dealing with images or natural language processing, where researchers often start training with simple samples progressing to complex ones. For instance, (Mousavi et al., 2022), designed a deep curriculum learning method for the classification of polarimetric synthetic aperture radar image in the order of easy to hard, which is evaluated by the patch complexity criterion, and achieved better accuracy than methods which consider samples in a random order during training. (Xu et al., 2020), proposed a method which is able to distinguish easy examples from difficult ones, and arrange a curriculum for language models, by reviewing the training set in a crossed way. In the application of robotics, researchers have focused more on applying curriculum learning to the generation of subgoals for traditional rigid manipulators. For instance, (Kilinc and Montana, 2020), trained a 7-DOF fetch robot for position to position tasks with a curriculum of intermediary imagined goals resulting in a high learning success rate. Zhou et al. (2021), also trained an industrial robot to overcome obstacles by generally increasing the size of obstacles. Mendoza investigated the curricula of a 6-DOF manipulator with Q-learning, such as the number of moving joints, joint velocities, or initial robot configurations (Diego Mendoza, 2017). The joints of these studied manipulators were directly driven by the corresponding actuators without any underactuated joints, and related research tends to be limited to simpler tasks such as reaching without considering orientation. In comparison, this study particularly focuses on more complicated manipulator structures and complex tasks, with regard to the positional and orientational control of a tendon-driven high-DOF underactuated manipulator.

### 2.2 Simulation of tendon-driven manipulator

To simulate a tendon-driven robotic system, the traditional approaches simulation often focus on constructing a mathematical model to map the tendon force/length to joint torque/angle, and applying these models to generate the robot motions. For instance, G. Borghesan et al. reported the development of a tendon-driven robotic finger by simulation, in which a finger model was constructed by mapping the joint torques to the tendon forces (Borghesan et al., 2010); Okoli et al. developed a cable-driven parallel robot simulation, in which the robot was investigated by moving an object using the tendon. The tendon length change and object twist was mapped using a Jacobian matrix (Okoli et al., 2019). Ko reported a tendon-driven gripper, constructing a hand model using relationships between tendon tension and joint torques (Ko, 2020). These mathematical-based approaches are often unsuitable for complex manipulator architectures, as (1) when some DOFs are underactuated or the relationship between joints and tendons is not a one-to-one correspondence, it is often difficult to find such a mathematical relationship; (2) the contacts between tendons and links are often changing depending on various postures, these contacts affect the manipulator actuation. Therefore, a mathematical model-free approach is beneficial to simulate such a tendon-driven underactuated manipulator.

### 2.3 Previous works of Robostrich arm

**FIGURE 1:**
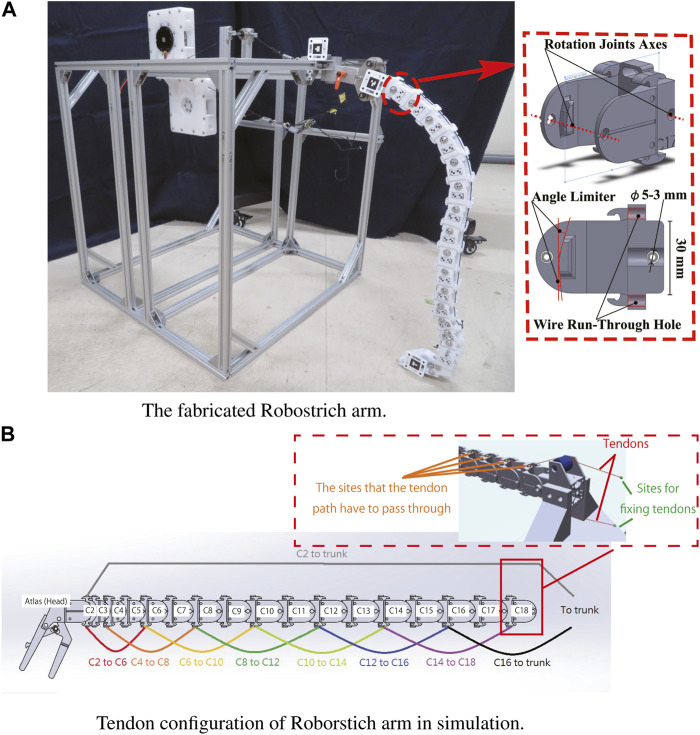
The fabricated Robostrich arm and tendon configuration in simulation. **(A)** The fabricated Robostrich arm. **(B)** Tendon configuration of Roborstich arm in simulation.

The Robostrich arm was designed with a focus on the dorsoventral motion of an ostrich neck to represent neck movement in the sagittal plane (Misu et al., 2022). The Robostrich arm comprises 18 rigid links with passive rotational joints, which is similar to the number of real ostrich vertebrae. The mean length of the links was designed according to an earlier anatomical report of a real ostrich and the sizes of the links were scaled to 75% (Dzemski and Christian, 2007). Additionally, the movable range of the joints was determined according to the flexibility measured from real ostriches in that earlier study. Table 1 lists the parameters of the Robostrich arm, and Figure 1A shows the Robostrich arm prototype. The links of the Robostrich arm were numbered from the C2 to C18, corresponding in order from the cranial to the caudal part of the ostrich vertebra. C2 denotes the link which is closest to the atlas (head). The length of the links in Table 1 is defined as the distance between adjacent rotational joint axes in the corresponding links. The masses in Table 1 are the masses of the bone parts, and the atlas mass includes the masses of the beak parts.

**TABLE 1:** Parameters of Robostrich arm. The links are numbered from C2 to C18 and counted from the cranial side to caudal side (see Figure 1B). C2 denotes the link which is closest to the atlas (head). The length is defined as the distance between the adjacent rotational joint axes in the corresponding link.

	Atlas (head)	C2	C3	C4	C5	C6	C7	C8	C9	C10	C11	C12	C13	C14	C15	C16	C17	C18
Dorsal angle limit (°)	1	12	18	20	28	31	33	35	28	29	30	27	24	22	19	18	18	18
Ventral angle limit (°)	1	12	16	18	25	30	32	30	26	25	18	15	12	8	6	6	6	6
Length (mm)		7.1	17.6	22.1	26.5	30.9	35.3	39.7	39.7	44.1	44.1	44.1	44.1	44.1	44.1	44.1	44.1	39.7
Mass (g)	83	5	7	8	10	11	12	13	13	14	14	14	14	14	14	14	14	13

In our previous study, two DC motors were used to connect four tendons to actuate the Robostrich arm, and a simple feed-forward strategy was used to control the manipulator. The preliminary results show that the head-up motion was successful only when the entire arm was maintained in a bar shape during rotation. However, the motion using this strategy limits the dexterity of the high-DOF manipulator. We considered the above problems as caused by a missing model to test its motion and control. However, constructing a mathematical model of such a complicated system is laborious and its parameters are not flexibly changeable, such as inertia moments and tendon attachment positions. Therefore, we aimed to solve the aforementioned issue using a curriculum-reinforcement learning approach.

## 3 Curriculum-SAC learning

We introduce a novel reinforcement learning-based approach for a complicated manipulator that can learn manipulation tasks. We adopted an architecture based on SAC reinforcement learning (Haarnoja et al., 2018) and curriculum learning (Bengio et al., 2009). In our tasks, SAC achieved the best performance among general reinforcement learning methods, as shown in the Supplementary Material. The neural network policy was trained using reinforcement learning to obtain higher rewards in the simulation environment.

### 3.1 Soft Actor-Critic

The learning objective of traditional reinforcement learning algorithms is to learn a policy that maximizes the reward of the trajectory.

$$\pi^{*}=\arg\max\underset{t}{\sum }E_{\left(s_{t},a_{t}\right)∼\rho_{\pi }}\left[r\left(s_{t},a_{t}\right)\right]$$ (1)

where *ρ*_*π*_(*s*_*t*_|*a*_*t*_) denotes the state-action marginals of the trajectory distribution induced by the policy *π*(*a*_*t*_|*s*_*t*_). In SAC, a maximum entropy reinforcement learning algorithm aims to learn a policy considering to maximize not only the reward of trajectory but also the entropy of each state, namely:

$$\pi^{*}=\arg\max\underset{t}{\sum }E_{\left(s_{t},a_{t}\right)∼\rho_{\pi }}\left[r\left(s_{t},a_{t}\right)+\alpha H\left(\pi \left(*|s_{t}\right)\right)\right]$$ (2)

where H denote the entropy term and *α* denote the temperature parameter that determines the relative importance between the rewards and entropy. Generally, the V function with entropy is referred to as the soft V function.

$$V_{\pi }\left(s_{t}\right)=E_{a_{t}∼\rho_{\pi }}\left[Q\left(s_{t},a_{t}\right)-\log\pi \left(a_{t}|s_{t}\right)\right]$$ (3)

and the Q function is defined by operator *T* ^*π*^:

$$T^{\pi }Q\left(s_{t}|a_{t}\right)=r\left(s_{t},a_{t}\right)+\gamma E_{s_{t+1}∼p}\left[V\left(s_{t+1}\right)\right]$$ (4)

Then, we use the soft policy evaluation, let *Q* ^*k*+1^ = *T* ^*π*^ *Q* ^*k*^, *Q* ^0^ = *S* × *A* → *R*, |*A*| < *∞*, where *k* → *∞*, *Q* ^*k*^ converges to a soft Q value. Because of the convergence of *Q* ^*k*^, policy *π* also converges to an optimal policy *π*∗ when |*A*| < *∞*.

SAC approximates the above formula and uses three parameters *φ*, and *θ*, *ϕ* to parameterize V, Q, and *π*, and update *φ*, *θ*, and *ϕ* in each step, respectively. Following this trick, we define the following objective:

$$\begin{array}{ll} J_{V}\left(\varphi \right) & =E_{s_{t}∼D}\left[\frac{1}{2}\left(V_{\varphi }\left(s_{t}\right)-E_{a_{t}∼\pi_{\phi }}\left[Q_{\theta }\left(s_{t},a_{t}\right)\right.\right.\right.\\ & \;-\left.{\left.\left.\log\pi_{\phi }\left.\left(a_{t}|s_{t}\right)\right)\right]\right)}^{2}\right] \end{array}$$ (5)

The soft Q-function is used to minimize the soft Bellman residual as follows:

$$\begin{array}{ll} J_{Q}\left(\theta \right) & =E_{\left(s_{t},a_{t}\right)∼D}\left[\frac{1}{2}Q_{\theta }\left(s_{t},a_{t}\right)-\left(r\left(s_{t},a_{t}\right)\right.\right.\\ & \;+\left.\gamma E_{s_{t+1}∼p}\left[V_{\bar{\varphi }}{\left(s_{t+1}\right)}^{2}\right]\right] \end{array}$$ (6)

Where D denotes the replay buffer, and *γ* denotes the discount factor. In practice, (Fujimoto et al., 2018) used the 2 Q functions *Q*_*θ*1_ and *Q*_*θ*2_. The objective function of the policies to be updated via minimization is as follows:

$$J_{\pi }\left(\phi \right)=E_{s_{t}∼D,\varepsilon_{t}∼N}\left[\alpha \log\pi_{\phi }\left(a_{t}|s_{t}\right)-Q_{\theta }\left(s_{t}|a_{t}\right)\right]$$ (7)

Where *ɛ* denote the input noise sampled from the Gaussian distribution N.

The temperature parameter *α* affects the exploration ability of the policy. We aim to achieve a flexible exploration of the strategy through dynamic automatic adjustment using the following optimal function:

$$J\left(\alpha \right)=E_{a_{t}∼\pi_{t}}\left[-\alpha \log\pi_{t}\left(a_{t}|s_{t}\right)-\alpha H\right]$$ (8)

### 3.2 Curriculum learning

We designed a curriculum course by adjusting the reward functions, initial states, and environmental setting of reinforcement learning, such that the agent can finally learn manipulation tasks and improve the performance from the traditional SAC approach.

In the first lesson, the reward function was defined as follows:

$$r_{1}=-\left(\|p_{\mathrm{head}}-p_{\mathrm{desire}}\|>d\right)$$ (9)

Where, *p*_*head*_ and *p*_*desire*_ denote the current and desired head positions, respectively, and *d* denotes the error tolerance of the head position. In this lesson, the agent is expected to learn a policy to reach the desired position without considering the attitude angle of the head.

In the following lessons, all reward functions are the same but have different initial states or environment settings.

$$\begin{array}{cc} r_{m2} & =-\lambda_{1}\left(\|p_{\mathrm{head}}-p_{\mathrm{desire}}\|>d\right)-\lambda_{2}\left(\|\theta_{\mathrm{head}}-\theta_{\mathrm{desire}}\|>\alpha \right)\\ r_{a2} & =-\lambda_{3}\|p_{\mathrm{head}}-p_{\mathrm{desire}}|-\lambda_{4}\|\theta_{\mathrm{head}}-\theta_{\mathrm{desire}}\|\\ r_{2} & =r_{m2}+r_{a_{2}} \end{array}$$ (10)

Where *θ*_*head*_ and *θ*_*desire*_ denote the current and desired attitude angles of the head, respectively. Weight *λ*_*i*_ determines the importance of the position and orientation of the head in the reward functions. In this lesson, we intended the agent to learn a more difficult policy so that it can reach the desired positions with a certain attitude angle of the head. In particular, in the second lesson, the agent starts learning in each episode with a fixed initial state and is expected to learn the more difficult policy with set position and head orientation. In the third lesson, we make the agent start learning using the final states of the last episode as the initial states, such that the agent can move among states continuously.

In the first three lessons, we intended the agent to acquire basic manipulation abilities to continuously track a set of target positions with specific orientations. In practical usage scenarios, manipulators are often combined with mobile platforms for broader applications (Osman et al., 2020). In contrast to the stationary case, in such scenarios, the influences of the environment should be learned, such as perturbations. Therefore, we introduced a subsequent lesson aimed at allowing the agent to acquire the advanced ability to respond to the environment. In other words, the training environment was modified by mounting a manipulator on a mobile platform.

### 3.3 Curricula reinforcement learning framework

The final algorithm is shown in Algorithm 1. The method alternates between collecting experience from the environment with the current policy and updating the function approximators in the order of curriculum settings using stochastic gradients from batches sampled from a replay pool. In our curriculum settings, each task is a sub-optimal solution for the next task; therefore, it is possible to help the agent to generate the action that maximizes the performance for a final task.

Algorithm 1 Soft Actor-Critic with Curriculum.

**Input:** *θ*_1_, *θ*_2_, *ϕ*
**1:** *θ*_1_ ←*θ*_1_, *θ*_2_ ←*θ*_2_
**2:** D←∅

**3: for** *each lesson* **do**
**4:** **for** *each iteration* **do**
5:  **for** *each environment step* **do**
**6:** $a_{t}∼\pi_{\phi }\left(a_{t}\;|\;s_{t}\right)$

**7:** $s_{t+1}∼p\left(s_{t+1}\;|\;s_{t},a_{t}\right)$

**8:** $D\leftarrow D\cup \left\{\left(s_{t},a_{t},r\left(s_{t},a_{t}\right),s_{t+1}\right)\right\}$

**9:**  **end**
**10:**  **for** *each gradient step* **do**
**11:** $\theta_{i}\leftarrow \theta_{i}-\lambda_{Q}{\hat{\nabla }}_{\theta_{i}}J_{Q}\left(\theta_{i}\right)$ for *i* ∈ {1, 2}
**12:** $\phi \leftarrow \phi -\lambda_{\pi }{\hat{\nabla }}_{\phi }J_{\pi }\left(\phi \right)$

**13:** $\alpha \leftarrow \alpha -\lambda {\hat{\nabla }}_{\alpha }J\left(\alpha \right)$

**14:** ${\bar{\theta }}_{i}\leftarrow \tau \theta_{i}+\left(1-\tau \right){\bar{\theta }}_{i}$ for *i* ∈ {1, 2}
**15:**  **end**
**16:** **end**
**17 end**
**Output:** *θ*_1_, *θ*_2_, *ϕ*

Where *θ*_1_, *θ*_2_, *ϕ* are parameters to be optimized, *D* denotes the replay buffer, *λ* is a parameter in the stochastic gradient method, and *π*, *a*, and *s* are introduced in Section 3.1.

## 4 Simulation platform

This section describes the simulation environment of the Robostrich arm. Based on this, two setups were prepared for training. In addition, the motion of the Robostrich arm is tested by manually controlling the tendon input in the simulation. Afterward, the workspace of the Robostrich arm is investigated.

### 4.1 Simulation environment of Robostrich arm

We used MuJoCo (Todorov et al., 2012) with the OpenAI gym toolkit (Brockman et al., 2016) to simulate the Robostrich arm. The assembled model of the Robostrich arm was created using CAD software and rendered in MuJoCo. The parameters of the Robostrich arm, such as the joint limitations and masses of the links, were manually edited according to Table 1. The tendons were defined with the attachment sites and were assigned to pass through the predefined wire run-through holes on the links (see Figure 1). We defined the general actuator properties and assigned them to act on the tendons. Therefore, the control of actuators can produce contraction of the corresponding tendons, thereby leading to the actuation of the manipulator. In this study, we refer to the ventral muscles of ostriches (Cobley et al., 2013) and modified the tendon arrangement from our previous work (illustrated in Figure 1B).

### 4.2 Experimental setups

To evaluate the performance of the Robostrich arm with curriculum-reinforcement learning, two situations were considered: (1) the Robostrich was mounted on an immobile frame; (2) the Robostrich was mounted on a commercially available quadruped robot (A1; Unitree Robotics, China).

**FIGURE 2:**
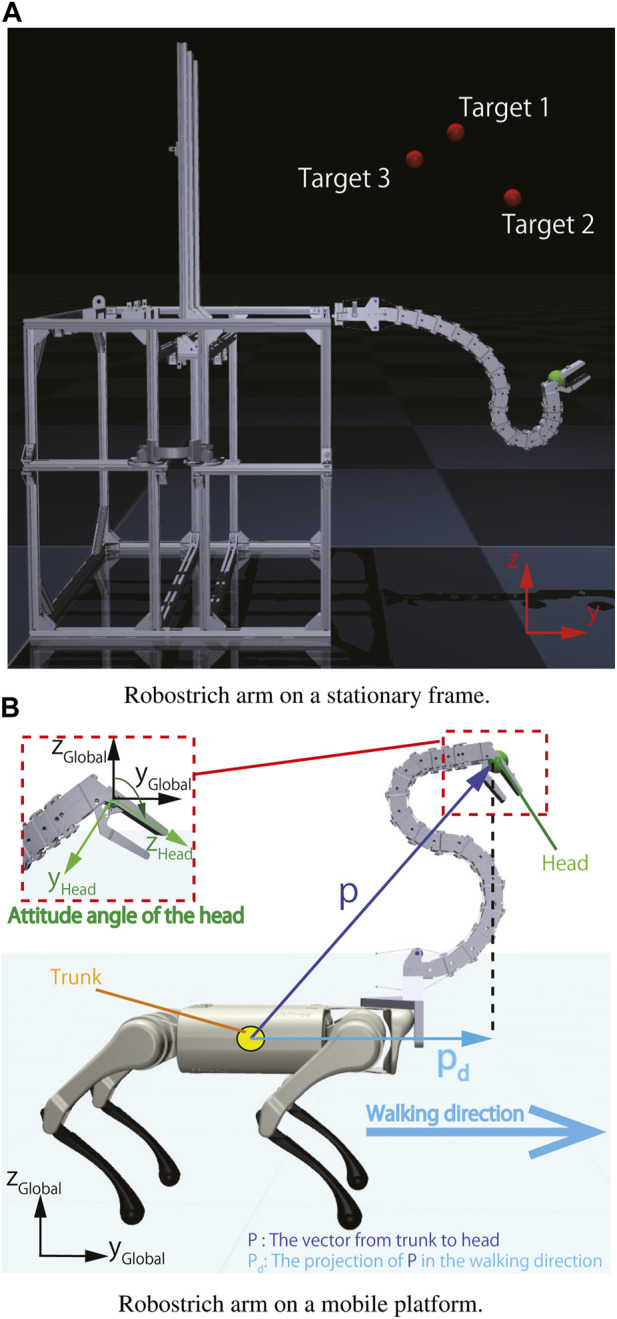
Environment setup. **(A)** Robostrich arm on a stationary frame. **(B)** Robostrich arm on a mobile platform.

In the first situation, we focused on a stationary situation in which the position of the Robostrich arm base was not changed, and the properties of the Robostrich arm and curriculum-reinforcement learning were investigated while not being affected by the disturbance of the base. In the second situation, vibrations were generated by the walk of the quadruped robot to serve as noise to the manipulator and we investigated the affected performance. In both situations, the timestep was set as 0.008 s. Figure 2 shows these two setups, and Table 2 listed the corresponding action and observation spaces. As summarized in Table 2, the total dimensions of the action space in both environments were identical, however, their total observation space dimensions differed. Additional dimensions were set owing to the locomotion of the quadruped robot. In particular, the walking pattern of the quadrupedal robot was determined by a set of motor data obtained from the walking task of the real A1 robot.

**TABLE 2:** Action and observation space of the simulation environments.

Environments	Action space (Dimension)	Observation space (Dimension)
The Robostrich was mounted to a stationary frame	Dorsal tendon (1D)	Head position (2D)
	Ventral tendons (8D)	Head velocity (2D)
		Joint angles (18D)
The Robostrich was mounted to a commercially available quadruped robot	Dorsal tendon (1D)	Head position (3D)
	Ventral tendons (8D)	Head velocity (3D)
		Joint angles (18D)
		Trunk position (3D)
		Trunk velocity (3D)

In the training stage, the target positions were randomly sampled from the workspace of the Robotstrich arm. The agent was thereafter trained by following the described curriculum learning process. In these policies, the positional and orientational error tolerances were set as ±0.05 m and ±5°, respectively. After training, all experiments were evaluated using the learned policies. The testing targets were selected based on the requirements of the manipulation tasks, and the desired attitude angle of the Robostrich head was set as 90° with respect to the global coordinate system. This implies that the orientation of the Robostrich head was maintained parallel to the ground during the experiments (see Figure 2B).

### 4.3 Movement test of Robostrich arm simulation

**FIGURE 3:**
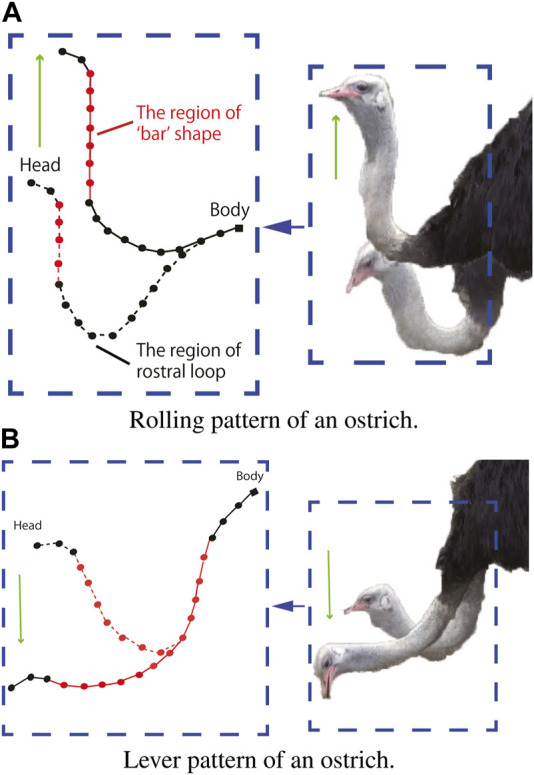
Motor pattern of an ostrich. **(A)** Rolling pattern of an ostrich. **(B)** Lever pattern of an ostrich.

**FIGURE 4:**
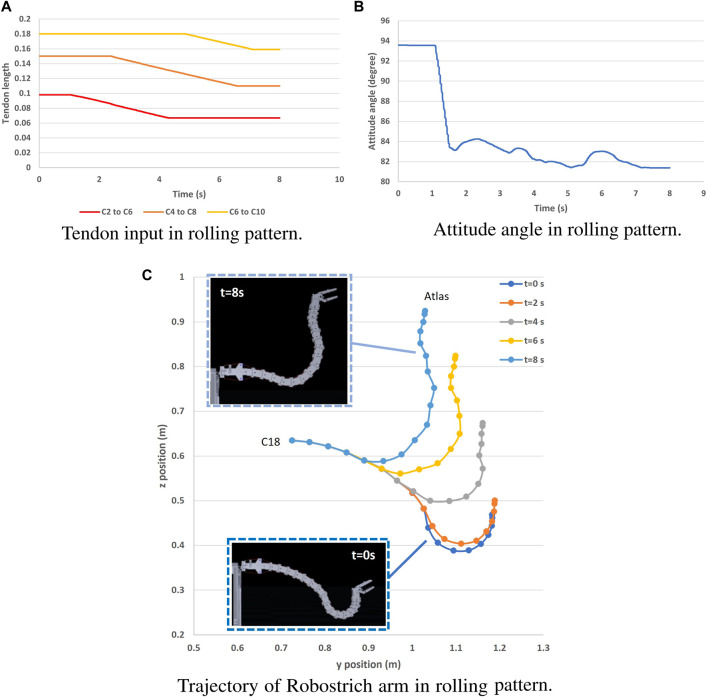
Rolling pattern movement of Robostrich arm on an immobile frame. **(A)** Tendon input in rolling pattern. **(B)** Attitude angle in rolling pattern.

**FIGURE 5:**
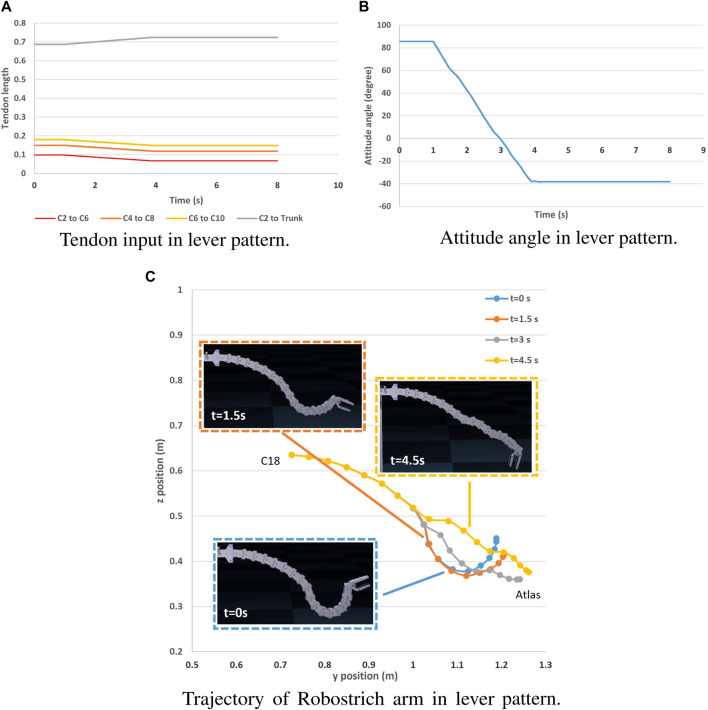
Lever pattern movement of Robostrich arm on an immobile frame. **(A)** Tendon input in lever pattern. **(B)** Attitude angle in lever pattern. **(C)** Trajectory of Robostrich arm in lever pattern.

The rolling and lever patterns are two representative motor patterns in the avian neck system that can be found during feeding or pecking. The rolling pattern can be roughly characterized as the transition of the rostral loop of vertebrae, leading to a change in the length of the cranial region of the neck, which can be partly maintained as a “bar” shape (van der Leeuw et al., 2001). This leads to the fact that the orientation of the head can be maintained during its positional movement. Instead of successive rotations, the lever pattern is characterized by simultaneous rotations in the rostral loop, and the angle between the bars of the caudal loop changes during neck movement, causing the caudal loop to widen during head protraction (the concept was illustrated in Figure 3). Referring to (Nakano et al., 2022) in which rolling and lever patterns were used to test the motions of a tendon-driven manipulator; therefore, we selected these two movements to test our simulation environment. We used the control method proposed by (Nakano et al., 2023) to manually control the contraction of the ventral tendons when maintaining the dorsal tendon length. The experiment was conducted using the immobile Robostrich arm environment. Figures 4, 5 show the tendon inputs, head orientations, and corresponding trajectory samples. It can be observed that the movement of both patterns are reasonably represented in the simulation.

### 4.4 Workspace of Robostrich arm

**FIGURE 6:**
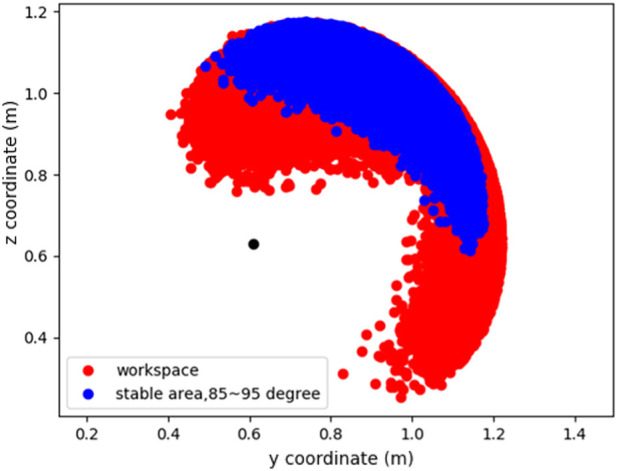
Workspace of Robostrich arm by sampling joint angles.

After confirming the motion, we determined the feasible area of the Robostrich arm to ensure that the target positions were selected in the workspace of the Robostrich arm. We randomly sampled the joint angles from the joint limitations of the Robostrich arm and plotted the corresponding head positions. Figure 6 shows the theoretical workspace of the Robostrich arm. The black point indicates the joint location between the C18 link and the frame, and the red range shows the area that the Robostrich head can achieve without considering the head orientation. The blue range shows the positions at which the head can achieve a head attitude angle of 90° ± 5°. This implies that if the target position is selected from the blue range, the head of the Robostrich arm can be maintained approximately parallel to the ground during head movement.

## 5 Experiments with curriculum-SAC learning

In the last section, simple motions were tested by manually controlling the tendons. To achieve more complicated manipulation tasks, the Robostrich arm was trained using the Curriculum-SAC learning approach. In the first experiment of this section, we evaluated the performance of learning methods in a stationary situation. In addition, we further studied the learning approach in the presence of noise with application scenarios (see Supplementary Material).

### 5.1 Experiment in immobile frame

**FIGURE 7:**
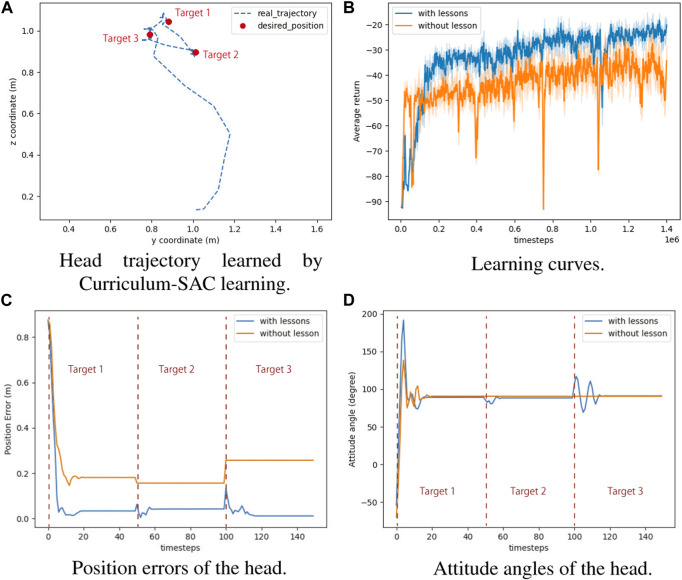
The results of reaching task with curriculum-SAC learning. **(A)** Head trajectory learned by Curriculum-SAC learning. **(B)** Learning curves. **(C)** Position errors of the head. **(D)** Attitude angles of the head.

In this experiment, we targeted a task in which the Robostrich head tracks a set of desired positions in order while keeping level with the ground. The Robostrich arm was trained using curriculum reinforcement learning as mentioned in the previous section, and the target positions were arbitrarily selected from the blue range in Figure 6; thus, the manipulator is theoretically capable of achieving the desired position and orientation (the attitude angle of the head was defined in Figure 2B). We compared the proposed learning method with a prior SAC reinforcement learning approach (Haarnoja et al., 2018), and the performance of the algorithms was evaluated at 140e^6^ steps because they approached asymptotic performance in our environment. The task of the experiment was to sequentially reach three target positions with the desired head orientation. Figure 7A shows the head trajectory of Robostrich arm using the learned Curriculum-SAC model. Figures 7B–D show the comparisons of the two algorithms regarding the learning curve, position error of the head, and head orientation, respectively.

According to the reward function of reinforcement learning, the agent optimizes both the angular and positional accuracies during motion. The results show that in the no-curriculum SAC, the agent exhibits better angular accuracy, but worse position accuracy. The Curriculum SAC allows the agent to learn the policy of optimizing the position accuracy through the pre-course, and based on this, the next course is learned, such that the position and angle accuracies are both within a reasonable range.

### 5.2 Experiments in mobile frame

#### 5.2.1 Head stabilization task

Vibration is often generated by the locomotion of a mobile platform, leading to a certain degree of noise in the control of the mobile manipulator. In this experiment, a quadruped robot was used to generate such vibration by controlling it while walking forward; hence the Robostrich arm vibrated depending on the gait of the quadruped robot. Curriculum-SAC learning performance was evaluated using a head-stabilizing task during walking. In addition, various trunk heights were set during walking to investigate the robustness of the controller in this task. In this scenario, walking gait and various trunk heights collaboratively contributed to vibrations in the Robostrich arm. In such a situation, the Robostrich arm must simultaneously control the position and orientation of its head. Therefore, controlling such a high-DOF tendon-driven underactuated manipulator is challenging. In the head stabilizing task, the agent was assigned to simultaneously: (1) maintain its head at a constant height to the ground; (2) maintain the attitude angle of its head near the desired orientation; and (3) maintain constant distance between the A1 trunk and the head of the Robostrich in the walking direction (*P*_*d*_ in Figure 2B). In the training stage, the agent was pre-trained using the first three lessons with the same training step as in the previous experiment when the quadruped platform was standing. Subsequently, an additional 400e^6^ training steps were performed with the walking of the quadruped platform.

**FIGURE 8:**
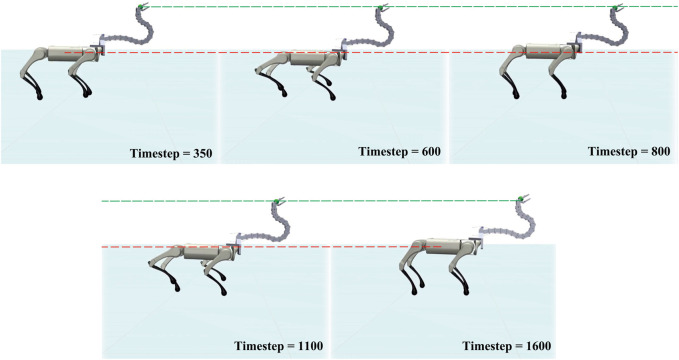
Sequence of “Head stabilization task”.

**FIGURE 9:**
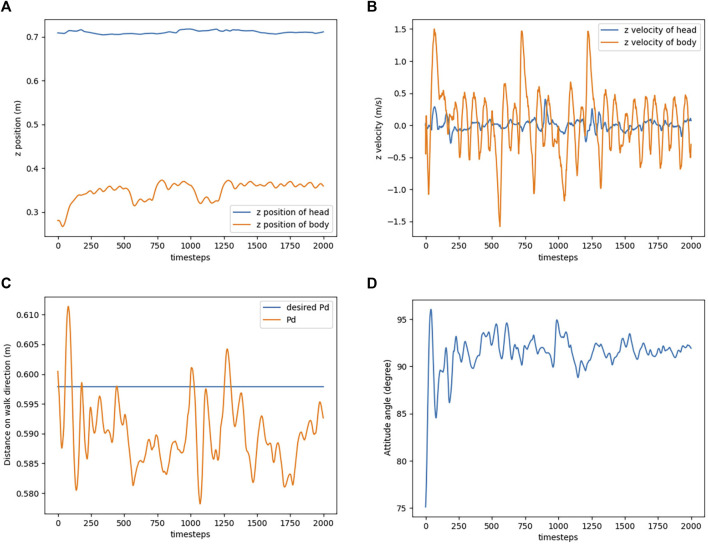
Simulation result of head stabilization task. **(A)** Head and trunk positions in *z* direction. **(B)** Head and trunk velocities in *z* direction. **(C)** Distance between the trunk and the head on walk direction. **(D)** Attitude angle of the head.

Figure 8 shows the movement of the agent during the experiment, and the corresponding results are shown in Figure 9. Comparing the position of the Robostrich head to the A1 trunk, it is apparent that the A1 trunk vibrated during walking, but a stable position can be found in the Robostrich head even though the height of the A1 trunk change (the timestep between the 500th and 750th, and the timestep between the 1000th and 1250th, in Figure 9A). In addition, the velocity of the Robostrich head is reduced. This implies that the proposed approach is robust against noise from mobile base vibrations. Although Figure 9C shows a certain degree of oscillation in the distance between the Robostrich head and A1 trunk in the walking direction, the mean values of the crests and troughs were approximated to the desired distance. In addition, the result in Figure 9D verified that the Robostrich arm learned to control its head orientation to approach the desired attitude angle, despite the change in A1 trunk height during locomotion.

#### 5.2.2 Reaching task in narrow space

Avoiding obstacles while reaching a target position is a useful feature of redundant manipulators. An experiment was conducted in a scenario where the mobile Robostrich arm passed through a narrow gap and reached a target position at the top of the gap. This is a difficult task for a traditional manipulator, but the additional DOF helps the Robostrich arm accomplish such a challenging task.

**FIGURE 10:**
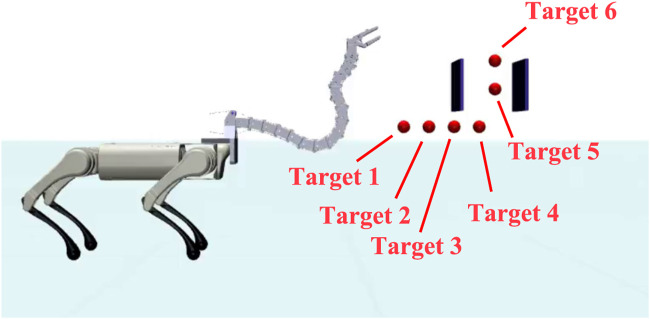
Experimental setup of “Reaching task in narrow space”.

The setup for this scenario is illustrated in Figure 10. In this reaching task, the quadruped platform was assigned to walk toward the narrow gap, and the head was assigned to reach target 6 (defined in Figure 10). Because the gap obstructs the Robostrich arm from directly reaching the target point, the arm must to move its head to the bottom of the gap in advance. Subsequently, the arm must lift its head vertically to pass through the gap. This action can be realized by continuously controlling the manipulator to track a set of predefined subtargets. As in the previous experiment, the agent was trained using the first three lessons and the fourth lesson with 140e^6^ + 400e^6^ training steps, which corresponded to the standing and walking situations.

**FIGURE 11:**
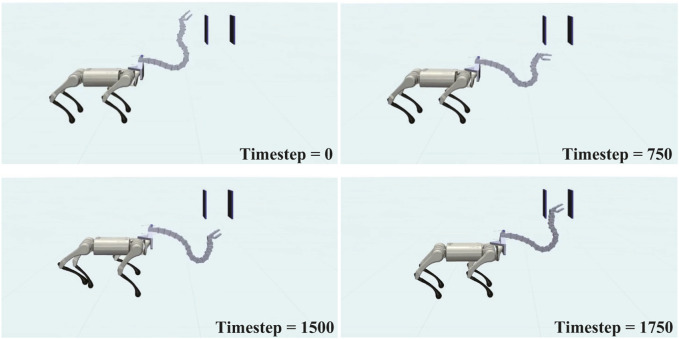
Sequence of “Reaching task in narrow space”.

**FIGURE 12:**
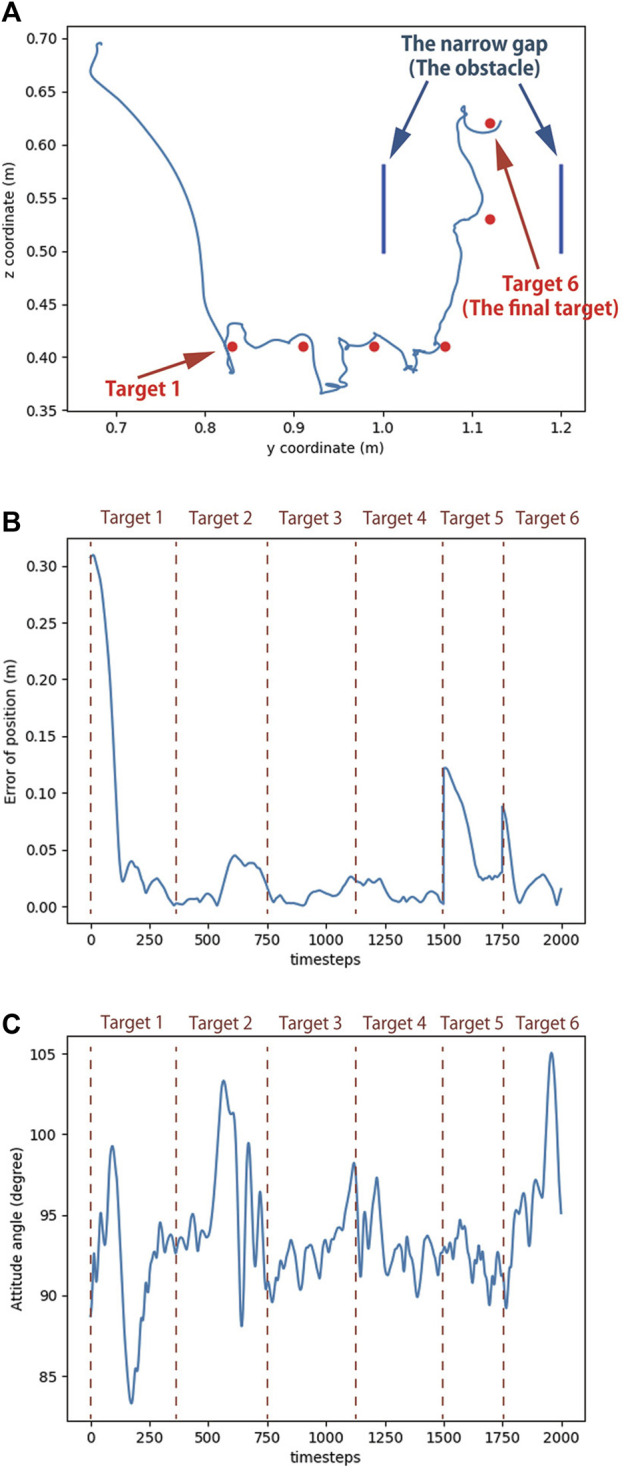
Simulation result of reaching task in narrow space. **(A)** Trajectory of Robostrich head. **(B)** Position error of the head. **(C)** Attitude angle of the head.

Figure 11 shows the movement of the robot during the experiment, and the corresponding head trajectory is shown in Figure 12A. The results indicate that the Robostrich head can approximately track the targets in the desired order (from targets 1–6), and the Robostrich arm can vertically raise its head to pass through the narrow gap, and finally reach target 6 at the top of the gap. The position error and attitude angle in the experiment are shown in Figures 12B,C, respectively. We found that the position error increased when the target position changed because the positional difference between the targets increased. We also found that such an error could be recovered immediately. Although we obtained high orientation errors in a few moments, the system rapidly recovered, and the head orientation was maintained within 90° ± 5°, which fulfills our error tolerance setup in reinforcement learning policies. These results verify that the agent can operate in a narrow space, which is useful for practical robotic applications.

## 6 Conclusion and future works

We aimed to develop ostrich-inspired manipulators to achieve dexterous manipulation and flexibility. A tendon-driven high-DOF underactuated manipulator was previously introduced inspired by the vertebral column of a real ostrich neck, known as the Robostrich arm. In this study, we proposed a curriculum reinforcement learning framework, to enable the Robostrich arm to gradually process from simple to more complex tasks. Four reinforcement learning lessons were defined depending on their complexities. These lessons can also be explained as courses from learning fundamental manipulation abilities, to obtaining advanced knowledge for specific tasks. A manual control experiment was conducted to confirm that the motion of the Robostrich arm can be reasonably represented in the simulation environment. In the experiments for training, we compared Curriculum-SAC learning with traditional SAC learning and found that the proposed learning method is effective in improving the accuracy of position control with a slight decrease in the performance of orientation control. However, the total reward of the proposed approach is higher than that of traditional SAC learning. Finally, we investigated the performance of Curriculum-SAC learning in the presence of noise by mounting the Robostrich arm on a quadruped platform. The simulation results demonstrate that the Robostrich arm can stabilize its head movement during walking and flexibly pass through a narrow gap to reach a target position at the top, despite perturbations are presented by locomotion. These results demonstrated the feasibility of curriculum-reinforcement learning for extended applications. Although the Robostrich arm was the focus of this study, our proposed learning method is also suitable for other complicated manipulator systems. In particular, more complex manipulation tasks can be trained by adding additional lessons.

In this study, the early demonstration of this approach was limited to simulation environments which are not a faithful representation of the real world. Some parameters should be further tuned depending on the real robot properties, such as friction coefficient, actuator gain, or sensor selection. To bridge the real world gap and transfer learned policies from simulation, some Sim2Real techniques are likely helpful to implement our approach in a real Robostrich arm, such as: Domain Randomization to randomize the friction and damping parameters in the simulation or add noise to the joint measurement (Tobin et al., 2017); Domain Adaptation to transfer the features from the source to the target domain to encouraging agents to adopt similar behaviors in a real environment (Christiano et al., 2016); Imitation learning to encourage agents to learn the policy for the real robot using the result of the expert policy from simulation, instead of directly applying it (Yan et al., 2017). In addition, only one tendon configuration was tested in the experiments; the comparison of various tendon configurations, and that between simulation and a real robot should be systematically investigated in the future. Regarding the learning method, although two application tasks were presented in this study, the number of tasks remained limited; therefore, verification with a wide range of tasks is necessary. Furthermore, it is necessary to clarify the network properties, such as (1) the factors affecting the control accuracy distribution, such as positions in the workspace, and (2) the relationship between control accuracy and various factors, such as various manipulator postures, different types of noise, or observations.

## Data Availability

The raw data supporting the conclusion of this article will be made available by the authors, without undue reservation.
