# Supplementary material for: Curriculum-reinforcement learning on simulation platform of tendon-driven high-degree of freedom underactuated manipulator
Source: Front Robot AI. 2023 Jul 12;10:1066518. doi: 10.3389/frobt.2023.1066518 (PMC10369056; doi:10.3389/frobt.2023.1066518)
Supplement: Supplementary file 1 [file DataSheet1.pdf]

Supplementary Material

1 SUPPLEMENTARY DATA

In this work, we focus on reinforcement learning methods, which can be divided into on-policy and off-policy according to sampling strategies. The on-policy sampling strategy makes it impossible to use past data, and the reinforcement learning method based on off-policy strategy has a wider application in the field of robotics. There are 3 general off-policy reinforcement learning algorithms: DDPG, TD3 and SAC. We test 3 algorithms, DDPG, TD3, SAC. The training convergence curves averaged over the 10 initial seeds are shown in Figure S1. The curves are smoothed with a rolling window average of 50 for clarity purposes. A comparison of the convergence curve is also reported for each algorithm individually. In these plots, the middle line represents the average reward over the 10 seed runs at each timestep and a shaded area is drawn at ± the standard deviation. Subsequently, these results are shown separately: DDPG, TD3 and SAC achieve the highest sample efficiency as their learning curves do not plateau after 500,000 timesteps. For DDPG and TD3, although algorithms plateau within 100000 steps, the average return becomes less. SAC achieves the best performance in both sample efficiency and training stability. Therefore, in this research, we take SAC as the benchmark for further reinforcement learning algorithm design.

2 SUPPLEMENTARY TABLES AND FIGURES

2.1 Figures

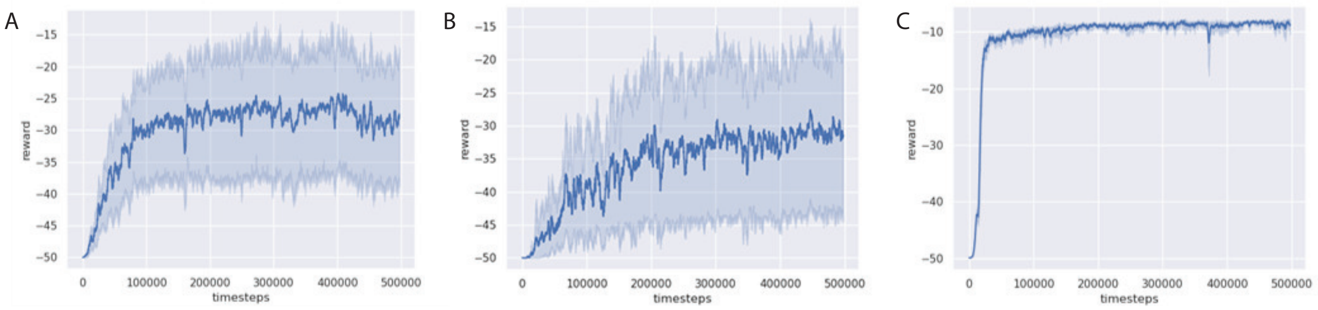

Figure S1. Comparison experiment for general Reinforcement Learning method. **(A)** DDPG. **(B)** TD3. **(C)** SAC.
